# Optoelectronic Properties of Atomically Thin Mo_*x*_W_(1−*x*)_S_2_ Nanoflakes Probed by Spatially-Resolved Monochromated EELS

**DOI:** 10.3390/nano11123218

**Published:** 2021-11-26

**Authors:** Mario Pelaez-Fernandez, Yung-Chang Lin, Kazu Suenaga, Raul Arenal

**Affiliations:** 1 Instituto de Nanociencia y Materiales de Aragon (INMA), CSIC-U. de Zaragoza, Calle Pedro Cerbuna 12, 50009 Zaragoza, Spain; mariopf@unizar.es; 2 Laboratorio de Microscopias Avanzadas, Universidad de Zaragoza, Calle Mariano Esquillor, 50018 Zaragoza, Spain; 3 National Institute of Advanced Industrial Science and Technology (AIST), Tsukuba 305-8565, Japan; yc-lin@aist.go.jp; 4 The Institute of Scientific and Industrial Research (ISIR-SANKEN), Osaka University, Osaka 567-0047, Japan; suenaga-kazu@sanken.osaka-u.ac.jp; 5 ARAID Fundation, 50018 Zaragoza, Spain

**Keywords:** band gap measurement, band gap engineering, optoelectronics, EELS, transition metal dichalcogenides, 2D materials

## Abstract

Band gap engineering of atomically thin two-dimensional (2D) materials has attracted a huge amount of interest as a key aspect to the application of these materials in nanooptoelectronics and nanophotonics. Low-loss electron energy loss spectroscopy has been employed to perform a direct measurement of the band gap in atomically thin Mo${}_{x}$W${}_{\left(1-x\right)}$S${}_{2}$ nanoflakes. The results show a bowing effect with the alloying degree, which fits previous studies focused on excitonic transitions. Additional properties regarding the Van Hove singularities in the density of states of these materials, as well as high energy excitonic transition, have been analysed as well.

## 1. Introduction

Atomically thin two-dimensional (2D) materials have been on the spotlight of modern research ever since the isolation of graphene in 2004 [1]. This ample interest comes for the most part from their appealing electronic, thermal and mechanic properties among others; as well as a vast number of potential and real applications [2,3,4]. Within 2D materials, layered transition metal dichalcogenide (TMD) semiconductors of the TX${}_{2}$ type (with T being a transition metal and X being a chalcogen) have attracted an important amount of research interest [5,6], given their interesting properties when it comes to optoelectronics [7,8,9,10,11], excitonics [11,12,13,14,15] and catalysis [16,17,18,19,20,21,22,23,24].

Concerning the electronic properties of 2D materials, tunable band gaps seem to have become crucial for the further development of electronic applications [24,25,26,27,28,29,30,31,32,33]. Most available 2D materials offer limited and rigid band gap values to work with (5.8 eV for monolayer BN [34,35,36,37] and 0 eV for monolayer graphene [38], for instance) and offer a limited versatility for electronic applications. TMDs offer a wider range of applications in this realm. Diverse approaches have been tried for band gap tuning in 2D materials, and TMDS have been no exception. Band gap tuning of 2D TMDs by means of functionalisation [30,39,40], dielectric screening [41], doping [42,43,44], straining [29,45] and phase engineering [46,47], alloying [48,49,50,51,52,53], as well as combinations of these methods [26], have been on the spotlight of 2D material research.

Alloying of materials with different band gaps has been a commonly used technique for band gap tuning in bulk materials. when it comes to 2D TMDs, even though bulk TMD alloys have existed for decades now, a wide array of 2D TMD alloys have been synthesised in the past decade [54], thanks to the development in synthesis techniques such as chemical vapor deposition [32], physical vapor deposition [55], chalcogen exchange [32] and colloidal solution synthesis [51,52]. Even so, only a small number of alloys has been achieved so far; most of them ternary alloys based on Mo, W, S, Se and Te [16,24,32,51,52,55,56,57,58,59,60], although new alloys with different transition metals have also been achieved [33,58,61,62], as well as quaternary alloys [17,63,64,65,66]. Novel optoelectronic applications have arisen from these new materials [66,67,68,69].

Within these alloys, Mo${}_{x}$W${}_{\left(1-x\right)}$S${}_{2}$ alloys have received an important amount of research interest, due to their being the first 2D TMD alloy to ever be synthesised and characterised [48,70,71], but also because of its various potential application regarding optoelectronics [53,72,73], hydrogen evolution reaction [23,74] and laser optics [75]. This has prompted an important amount of research efforts with the objective of characterising and modelling the behaviour of these alloys, especially when it comes to their optoelectronic properties [25,48,70,76,77,78].

However, the studies concerning the band gap of Mo${}_{x}$W${}_{\left(1-x\right)}$S${}_{2}$ alloys are based on photoluminescence (PL) and scanning tunneling spectroscopy (STS) experiments, and have only focused on the low energy empty states of these materials [16,48,77,78,79]. These are related to the band gap of a specific material, but they do not conform a direct measurement of said band gap, even though it has been discussed as such in the literature [80]. In this sense, one of the main focus points of this study is performing a direct measurement of the band gap for the different alloys presented.

This study aims as well for a detailed and direct characterization of optoelectronic properties in atomically thin Mo${}_{x}$W${}_{\left(1-x\right)}$S${}_{2}$ alloys as a function of their alloying degree. Additionally, STEM-EELS offers the possibility to perform these studies at the nanoscale while being able to calculate the thickness of the measured sample, therefore comparing the results obtained not just by the alloying degree of regions being sampled but also their number of layers.

## 2. Methods

### 2.1. Sample Preparation

Mo${}_{x}$W${}_{\left(1-x\right)}$S${}_{2}$ single crystals have been synthesised by means of chemical vapor transport (CVT), as done in previous works [48,70,71,72,81]. Different precursor ratios have been used to obtain different final alloying degrees. After synthesis, the crystals have been mechanically exfoliated, and the alloy flakes have been transferred to TEM grids.

### 2.2. Sample Characterisation

Once the samples have been prepared, for each one of them areas presenting a low number of layers have been identified and selected. This identification has been performed by means of optical and low-magnification TEM images such as the ones presented in Appendix A. These areas were later identified for subsequent spectroscopic measurements.

Initial characterisation of the different alloys has been performed using two STEMs, a JEM-ARM200F with a CEOS corrector was operated at 80 kV and a Jem-2100F with a DELTA corrector was operated at 60 kV. All monochromated STEM-EELS studies performed for this study has been carried out using a ThermoFischer Titan probe-corrected microscope working at 80 KV. The microscope is equipped with a Gatan Energy Filter (GIF) Tridiem 866 ERS and a monochromator that combined grant a resolution in energy of about ∼180 meV.

**Figure 1:**
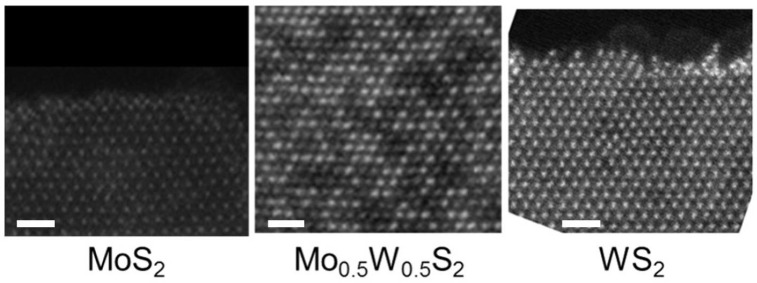
HAADF-STEM micrographs of MoS${}_{2}$ (**left**), Mo${}_{0.5}$W${}_{0.5}$S${}_{2}$ (**center**) and WS${}_{2}$ (**right**) samples. The difference in contrast between the Mo and S atoms can be seen in the micrograph corresponding to Mo${}_{0.5}$W${}_{0.5}$S${}_{2}$ in the form of brighter and dimmer spots. This is the difference in contrast that has been used to estimate the alloying degree as stated in the literature [71]. Scale bar: 1 nm.

Initial HRSTEM characterization has been performed in alloy monolayers. Figure 1 shows three different HRSTEM high-angle annular dark field (HAADF) micrographs corresponding to three monolayers of MoS${}_{2}$, Mo${}_{0.5}$W${}_{0.5}$S${}_{2}$ and WS${}_{2}$ samples, respectively. As it can be seen in these micrographs, as well as previous works [48,71], these Mo${}_{x}$W${}_{\left(1-x\right)}$S${}_{2}$ alloys present a hexagonal layered structure, corresponding to the 2H phase in their precursors MoS${}_{2}$ and WS${}_{2}$.

The alloying degree of these alloys has been directly determined from the proportion of Mo and W in these micrographs, deduced from the different contrasts among Mo, W and S atoms due to their difference in atomic number [71]. Five Mo${}_{x}$W${}_{\left(1-x\right)}$S${}_{2}$ samples were used in this study; each one with a different alloying degree: x = 0, 0.3, 0.5, 0.7 and 1, respectively.

**Figure 2:**
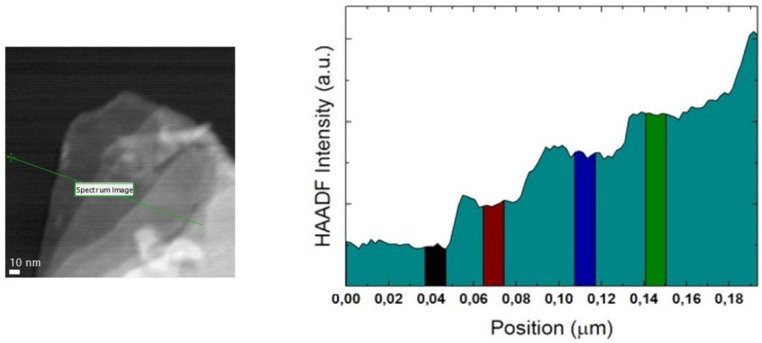
Characterisation of atomically thin Mo${}_{0.5}$W${}_{0.5}$S${}_{2}$ flake. **Left**: HAADF-STEM micrograph. The location of the STEM-EELS spectrum line is marked using a green line. **Right**: HAADF-STEM intensity along the spectrum line. Region in black shows the spectra appertaining to aloof spectroscopy. Highlighted flat windows show areas with the same number of layers, where spectra have been integrated.

For each alloy, In each few-layer region of interest, HAADF-STEM micrographs have been taken to determine where to perform the STEM-EELS studies. In these locations, low-loss EEL spectra were recorded using the spectrum-line mode [82]. An HAADF-STEM intensity profile was recorded simultaneously as well. An example of a spectrum-line scan, as well as its corresponding HAADF-STEM intensity profile, is shown in Figure 2.

### 2.3. Data Treatment

For each one of the spectrum profiles, several spectra have been integrated. On the one hand, 6 spectra have been integrated right below the onset for the HAADF-STEM intensity. These integrated spectra account for the aloof spectroscopic analysis needed for band gap estimation. On the other hand, regions have been identified along the profile where the intensity plateaus. This, along with their corresponding HAADF-STEM micrograph, has served to identify regions in the intensity profile where thickness is constant. In these regions, spectra have been integrated over a window between 10 and 30 nm wide, integrating 4 to 6 spectra to improve the signal-to-noise ratio (SNR). Spectra close to the edges of the different plateaus have purposefully been taken out of the integrated spectra to minimise plasmonic contributions, as they have been seen for MoS${}_{2}$ [83,84,85]. A depiction of these flat areas can be seen in Figure 2. For every integrated spectrum, the thickness of the spectra has been estimated using the log-ratio method [86]. Further insight on this estimation, can be seen in Appendix A.

For each integrated spectrum, after zero loss peak (ZLP) extraction, two different types of analysis have been performed in each spectrum, focusing respectively on the band gap of the sample on one hand and on the rest of optoelectronic features on the other hand. No analyses could be performed on the nature of the A and B excitonic peaks due to the energy resolution in these works not being able to discern both features, but given these two excitonic features are the main influence on the band gap of the samples, it is sensible to assume their behaviour as a function of alloying degree will be similar to that of the band gap.

Regarding the band gap analysis, focused in the spectral region between 1.5 and 2 eV, initially a Richardson-Lucy deconvolution has been performed in order to increase the SNR in the spectra [35,87,88]. The deconvoluted spectra have been linearly fitted over a window of 0.1 eV situated in the band gap region. The x-intercept value of said fit has been taken as the measured band gap value of the sample in the region [35,88].

As for the rest of the features in the low-loss EEL spectra, a triple Gaussian fit of each spectrum has been performed in an energy window between 2.5 and 6 eV in order to determine the values of the C excitonic feature as well as the α and β Van Hove features (see next section). The full-width at half maximum of the Gaussian fit related to the β Van Hove features has been constrained in order to keep it coherent with the data analysed, as it is discussed in Appendix A. The presence of an excitonic feature in this spectral region is discussed in Appendix A as well.

## 3. Results and Discussion

### 3.1. Integrated EEL Spectra; Features and Interpretation

In order to better interpret the gathered EEL spectra in these alloys, it is important to delve deeper into their optoelectronic properties. The main phenomena that rules over the electronic band structure of the samples, and in turn its band gap, are excitons. The dielectric confinement in TMDs incites the formation of highly stable, strongly bound excitons at low energies [12,58]. Due to high spin-orbit coupling, there is a spin splitting in the valence band, giving rise to two excitonic features in the EEL spectra at low energy, called A and B respectively. A third excitonic feature, called C, has been reported in the literature both from an experimental and a theoretical point of view [89,90,91]. As it can be seen in Appendix A, the data gathered in these works fits the presence of this excitonic feature in these samples, and its analysis is included in the optoelectronic characterisation of said samples.

An additional property of interest in these materials when it comes to their optoelectronic properties is the existence of discontinuities in their density of states (DOS). This kind of discontinuities are called Van Hove singularities, and their effect can be seen in the EEL spectra as features around this discontinuity in the DOS, since these discontinuities serve as triggers for transitions at a very specific energy. In the case of the materials that we study in these works, they both exhibit two distinct Van Hove singularities, which in turn produce two separate features in the EEL spectra, denominated as α and β respectively. These features have been previously identified in EEL spectra for MoS${}_{2}$ [92,93,94].

It is important to notice that the intensity in the β Van hove region for the aloof spectra is noticeably lower in comparison with the rest of the spectra. Our hypothesis for this behaviour is that it could be likely related to some volume/bulk (even for the case of monolayer case) contribution on these involved electronic features. For instance, similar behavior has been observed in the case of single-walled boron nitride nanotubes [35].

**Figure 3:**
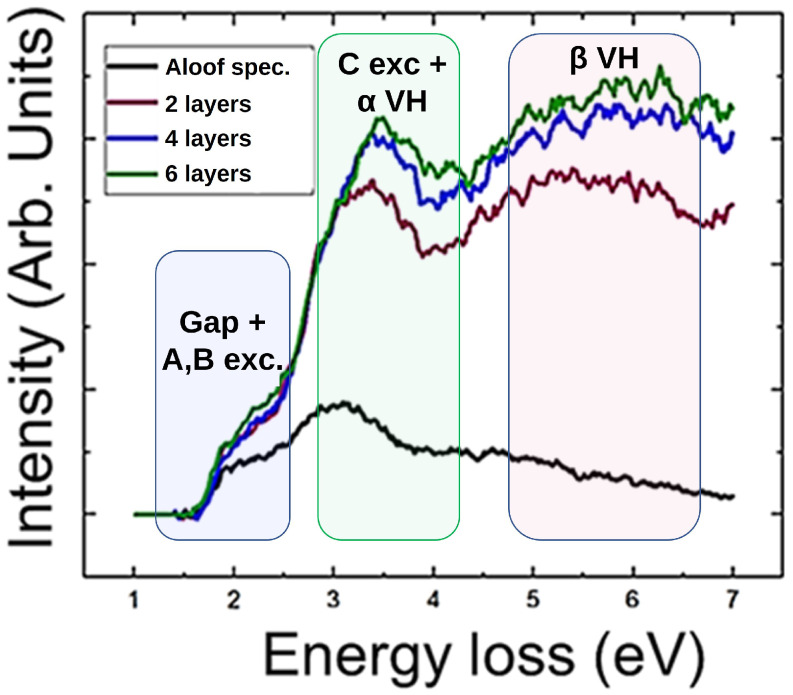
Integrated spectra from the regions highlighted in Figure 2 of a Mo${}_{0.5}$W${}_{0.5}$S${}_{2}$ flake. Same colours are assigned to the integrating window and integrated spectra for clarity. Three different regions of interest are highlighted: The one corresponding to the study of the band gap and the presence of the A and B excitonic features (blue), the one corresponding to the C excitonic feature, overlapped as well with the α van Hove feature (green) and the one corresponding to the β van Hove feature.

Figure 3 shows the integrated spectra from the regions highlighted in Figure 2, along with the regions of interest with respect to these studies.

As it can be seen, the C excitonic feature and the α van Hove feature appear to be overlapped on the same energy window. However, it has been proven (see Appendix A) that both features can be distinguished from one another.

### 3.2. Band Gap

**Figure 4:**
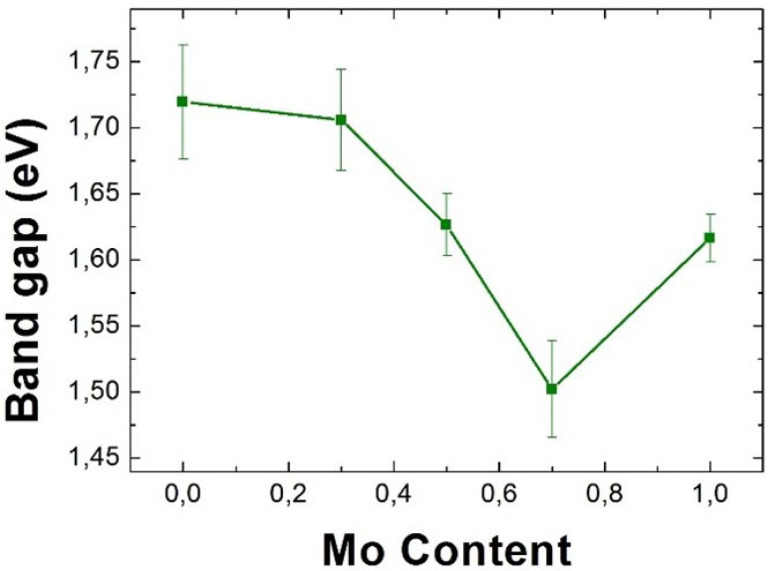
Band gap as a function of the Mo content for Richardson-Lucy deconvoluted EELS spectrum-line. The error bars represent the statistical deviation among measurements.

The results concerning the band gap measurements as a function of Mo content (alloying degree) can be seen in Figure 4.

These results represent the first direct measurement of the optical gap of such nanomaterials to the best of our knowledge. Generally speaking, the preferred technique of choice for the measurement the optical gap of a bulk material is optical absorption [95], which probes the frequency dependence of the imaginary term in the dielectric function $\left(\varepsilon \left(\omega \right)\right)$ [96]. On the other hand, EEL spectra are proportional to the energy loss function $\left(Im\left(-1/\varepsilon \left(\omega \right)\right)\right)$ [97]. In bulk materials and penetrating geometry (when the electron is going through the sample), provided we neglect surface loss contributions, this term for the energy loss function can be expressed as:

(1) $$Im\left(-1/\varepsilon \left(\omega \right)\right)=\varepsilon_{2}/\left(\varepsilon_{1}^{2}+\varepsilon_{2}^{2}\right)$$

where $\varepsilon_{1}$ and $\varepsilon_{2}$ are the real and imaginary parts of the dielectric function, which Kramers-Kronig transformations allow to determine from the measured energy-loss function [97,98]. However, this is not the case for thin 2D materials, where the surface effects are much more prominent.

One of the many benefits of STEM-EELS is that it allows a different acquisition mode, non-penetrating “aloof spectroscopy” [99]. In this type of spectroscopy, where the electron beam is set at a grazing position from the nanostructure, it is possible to use the continuous dielectric model (CDM) to delve into the optical responses using EELS [35,88,100,101,102,103,104,105]. It has been shown that, for different nanostructures, in aloof EELS the spectrum is a weighted sum of the imaginary parts of the multipolar polarizabilities γ [104,106]. Provided that few-layer TMD flakes can be considered in the strong-coupling regime of the CDM, the imaginary part of the polarizability can be written for a slab as:

(2) $$\;Im\left(\gamma \left(\omega \right)\right)\;=\;(Im\left(\varepsilon_{⊥}\left(\omega \right)-1/\varepsilon_{\|}\left(\omega \right)\right))$$

where $\varepsilon_{⊥}$ and $\varepsilon_{\|}$ are the components of the dielectric tensor of an alloy sheet that are perpendicular and parallel to the anisotropy axis, respectively.

In the case of MoS${}_{2}$ and WS${}_{2}$, it has been previously studied that the response of $\varepsilon_{\|}$ begins at about 3 eV, therefore it is possible to say that, below this energy, the energy loss function is proportional to $\varepsilon_{⊥}$ [103,104,106]. This is what has allowed for the direct measurement of the optical band gap in these samples, which has been taken as the inset of the energy loss ), as it has been previously done for the case of boron nitride nanotubes [88].

Much like previous works on the excitonic behaviour of these alloys [48,57,77,78,79,80,107], as well as recent modelling [25], the results on the value of the the band gap for different alloying degrees show a bowing effect as a function of the alloying degree. This bowing effect has been found to originate from the different variation of the valence and conduction bands with the alloying degrees. Density functional theory (DFT) calculations have shown that, whereas the valence band variates linearly, the conduction band varies non-linearly with the alloying degree [48].

The band gap values that have been found are slightly lower than those shown in the literature for photoluminescence (PL) studies, but it is coherent, since generally the value given for the band gap for these materials is the value of the A exciton peak, which dominates the band gap but is slightly higher in energy. It has not been possible, however, to discern if the band gap shifts in a particular way with the number of layers for each individual alloy. Discussion on this topic can be seen in Appendix A.

### 3.3. C Excitonic Feature

**Figure 5:**
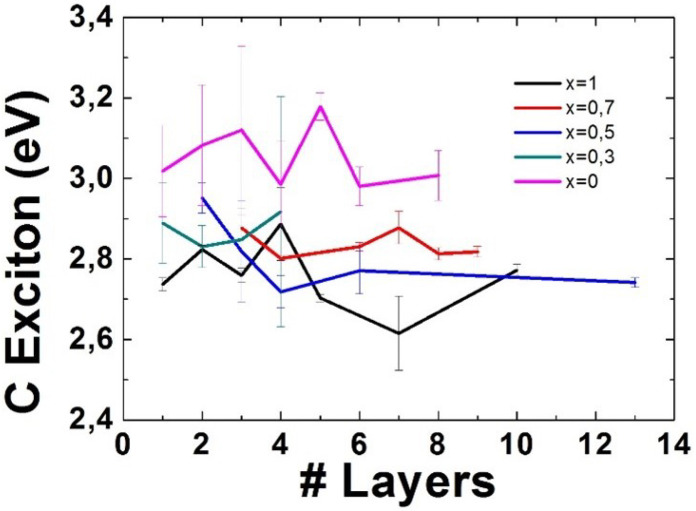
Position of the Gaussian fit corresponding to the C excitonic features for different number of layers and different alloying degrees. The error bars represent the error in the Gaussian fit for those layer numbers where only one measurement could be taken, and the standard deviation among measurements for those where several measurements could be taken.

The average value of the C excitonic features for each number of layers and each alloying degree can be seen in Figure 5. The results show a slight tendency from the C excitonic features to decrease with the number of layers for each specific alloying degree. This behaviour is more evident for some of alloying degrees. Regarding the behaviour of the C exciton with respect to the alloying degree, the position of these C excitonic features seems to decrease with the alloying degree as well. It is worth to remark the most notable difference in these values among alloying degrees, which occurs for WS${}_{2}$.

### 3.4. Van Hove Features

**Figure 6:**
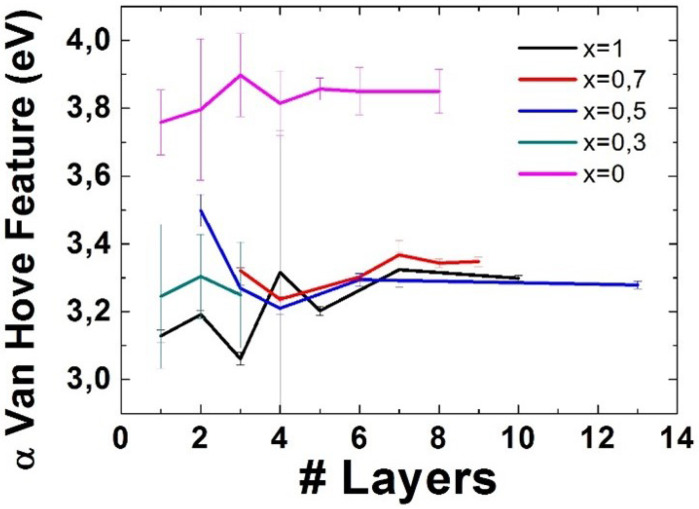
Position of the Gaussian fit corresponding to the α Van Hove feature for different number of layers and different alloying degrees. The error bars represent the error in the Gaussian fit for those layer numbers where only one measurement could be taken, and the standard deviation among measurements for those where several measurements could be taken.

Results regarding the position of the Gaussian fit assigned to the α Van Hove feature are shown in Figure 6.

Even though the results for low number of layers show a small decrease for x = 0.5, there is a general, albeit small, increase of the α Van Hove feature with the number of layers. As for the variation of this feature with the alloying degree, it seems to go down as the alloying degree goes up. Once again, the great difference in the value for this feature between WS${}_{2}$ and the rest of the alloys is also worth mentioning.

**Figure 7:**
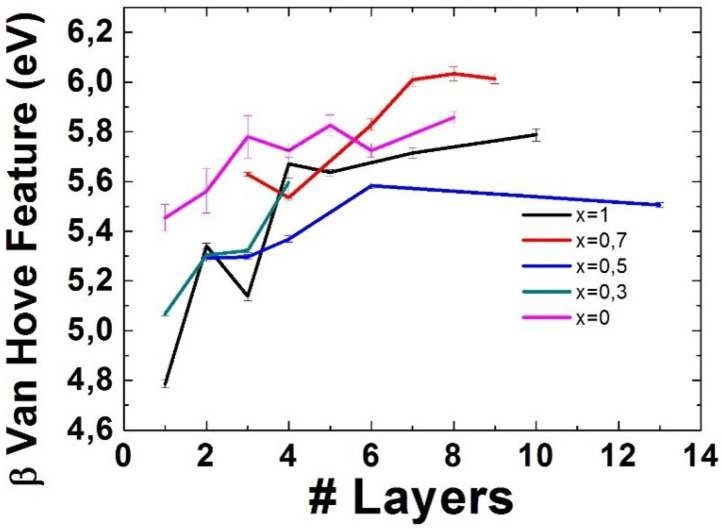
Position of the Gaussian fit corresponding to the β Van Hove feature for different number of layers and different alloying degrees. The error bars represent the error in the Gaussian fit for those layer numbers where only one measurement could be taken, and the standard deviation among measurements for those where several measurements could be taken.

Finally, results concerning the behaviour of the position of the Gaussian fit assigned to the β Van Hove feature can be found in Figure 7.

Results show a very clear increase in the position of this feature as the number of layers increases. However, it is not as easy to discern the behaviour of this feature as a function of the alloying degree of the material. This can be due to the analytic constrains set for this fit to keep the rest of the analysis consistent (see Appendix A). The raw results on each individual measurement of all features in the low-loss EELS region can be seen in Appendix A as well.

## 4. Conclusions

A direct measurement of the optical band gap of several Mo${}_{x}$W${}_{\left(1-x\right)}$S${}_{2}$ alloys is reported by means of non-penetrative low-loss EELS analyses. This band gap oscillates between 1.5 (reached at x = 0.7) and 1.72 eV (reached at x = 0), showing a bowing effect that is coherent with both previous experimental and modelled results [48]. The A and B excitons are expected to follow the same behaviour.

The behaviour of the C excitonic feature, due to several excitonic transitions shows a slight tendency from the C excitonic features to decrease with the alloying degree, and within the same alloying degree it seems to have a slight decreasing tendency as the number of layers goes up.

As for the α and β van Hove Features, a decreasing tendency can be found for the α feature as the alloying degree increases, whereas no clear tendency can be found for the β feature. As for the behaviour with respect to the number of layers, they both seem to increase in energy with thickness, albeit not in a uniform way.

This detailed optoelectronic characterisation of an atomically thin tunable sample composed of transition metal dichalcogenides will be of great use for future nanooptoelectronic and nanophotonic applications of these materials.

## Figures and Tables

**Figure A1:**
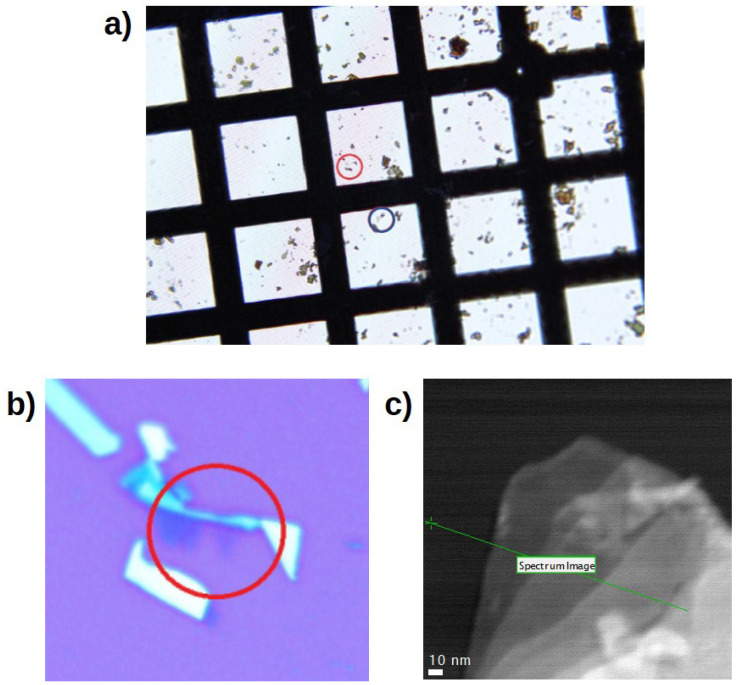
Optical microscopy and STEM characterization of atomically thin regions of interest. (**a**) Low-mag optical microscopy image showing two regions of interest where particularly thin alloy flakes have been found (red and blue circles, respectively). (**b**) High-mag optical microscopy image showing the region of interest seen in the red circle of the low-mag image. (**c**) STEM micrograph of the same alloy flake shown in the optical micrographs.

**Figure A2:**
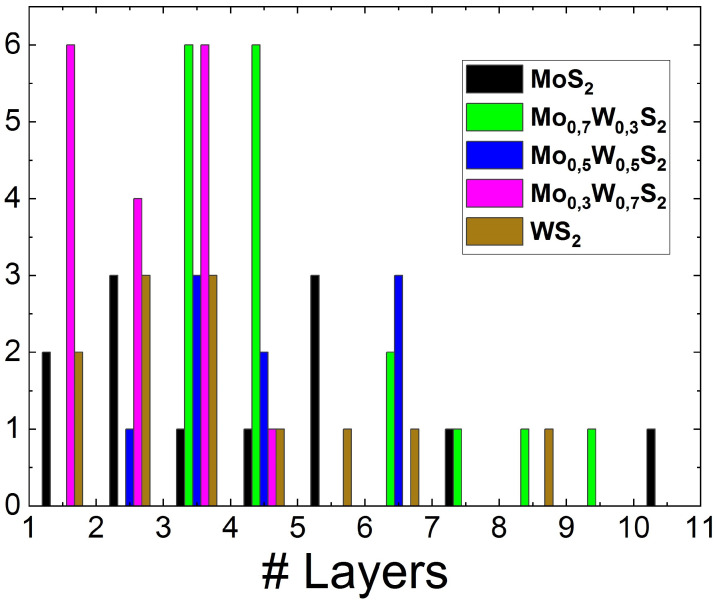
Histogram showing the number of layers measured for each region of interest in each alloying degree (Mo content).

**Figure A3:**
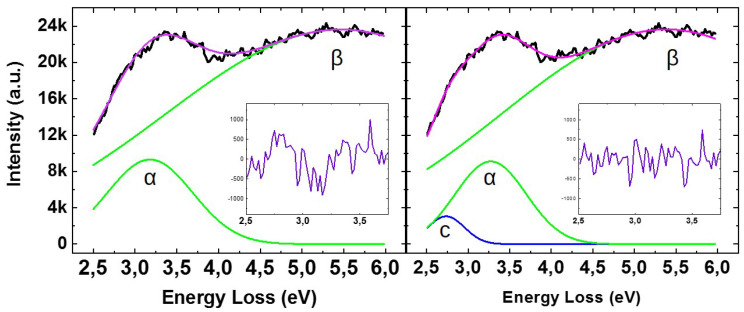
Comparison of a double and triple Gaussian fit on the 2.5–6 eV energy window for an EEL spectra. Inset: Residuals from the Gaussian fit.

**Figure A4:**
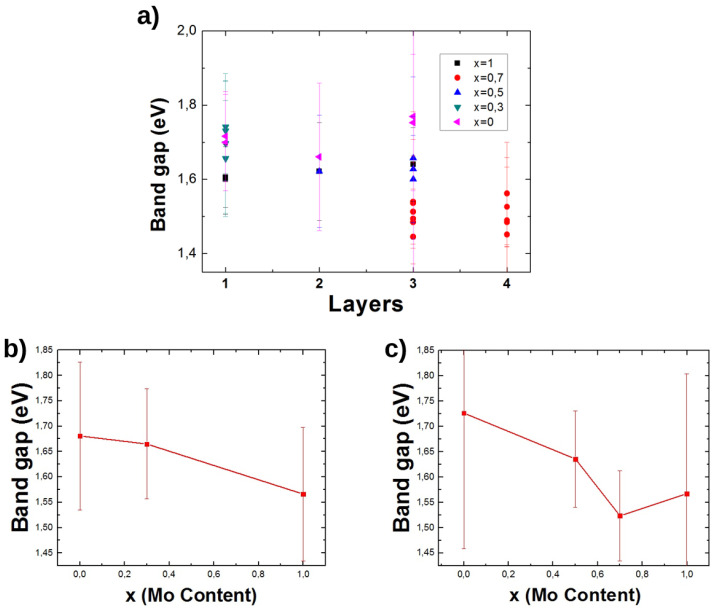
Additional results concerning the alloy band gap. (**a**) Raw values for the band gap measurements in these studies. Error bars are taken as the error in the onset of their corresponding linear regression. (**b**) Average results for alloy monolayers. (**c**) Average results for alloy trilayers.

**Figure A5:**
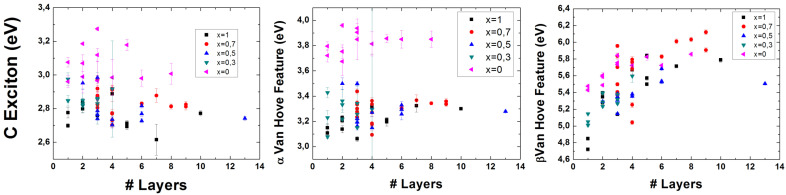
Raw results of the analyses in these works. Left to right: C exciton, α and β van Hove features.

## Data Availability

Data available by request to the corresponding author in this article.

## Abbreviations

The following abbreviations are used in this manuscript:

PL	Photoluminescence
HRSTEM	High-resolution scanning transmission electron microscopy
HAADF	High-angle annular dark field
DFT	Density functional theory
EELS	Electron energy loss spectroscopy
STS	Scanning tunnelling spectroscopy

## Appendix A. Additional Information of Interest to These Works

### Appendix A.1. Optical Characterisation

Figure A1 shows examples of the optical microscopy and STEM images used to find regions of interest with flakes presenting a few layers in the different alloys. Optical microscopy has been used to identify regions with thin flakes, which have then been identified by means of STEM imaging.

### Appendix A.2. Thickness Measurements

A log-ratio method has been employed for the estimation of the thickness in the different alloy flakes. As it has been shown in the literature [86], besides other experimental parameters, this analysis need the effective atomic number Z${}_{eff}$ of the sample. Since the alloy flakes have been shown to be homogeneous in composition, with no clustering of any kind [71], Z${}_{eff}$ can be calculated as a function of the alloying degree and the atomic numbers of Mo, W and S:

(A1) $$Z_{eff}=\frac{x\times N_{Mo}+\left(1-x\right)\times N_{W}+2\times N_{S}}{3}=\frac{x\times 42+\left(1-x\right)74+2\times 16}{3}=\frac{106-32x}{3}$$

By dividing the thickness of the sample by the interlayer distance in MoS${}_{2}$ and WS${}_{2}$ (∼6.5 Å [108]), the number of layers of each region has been found. A histogram of the number of measurements performed for each number of layers in each sample can be found in Figure A2.

### Appendix A.3. C Exciton and Van Hove Features Analysis

When it comes to the analysis of the C exciton and Van Hove features, a multipeak Gaussian fit approach has been used. However, some restraints have been needed to keep the analysis consistent. Since the feature corresponding to the β Van Hove feature is way more intense than the rest of the features on this window, the behaviour of the Gaussian fit for this feature dominates the analysis on the whole spectral window.

In this sense, we have assumed to have a Gaussian fit for this feature that does not change in width with the number of layers, since there was a clear correlation between the changes in width of the Gaussian fit for the β Van Hove feature and the shift in energy for the other two features in this window. For this, a preliminary analysis has been set in place to gather some insight on the width of this feature, and a second fit has been performed constraining the width of the β Van Hove feature Gaussian to the mean value obtained in the preliminary analysis for that particular alloy.

The fit used also shows the importance of the C excitonic feature, not just from an optoelectronic point of view, but also for the correct analysis of the α van Hove features, since it is evident from the residuals of the analysis that a double Gaussian has trouble correctly fitting the spectra. Figure A3 shows the analysis results for a double Gaussian and a triple Gaussian fit in the energy window from 2.5 to 6 eV.

### Appendix A.4. Raw Results

The raw results for the values of the bandgap, as well as the average estimation that has been able to be done for the case of monolayer and trilayer samples, can be seen in Figure A4.

Results show that, even though it is not possible to discern a general behaviour of the band gap with the number of layers, both monolayer and trilayer measurements show the same behaviour as a function of the alloying degree as the general average band gap value.

Figure A5 shows the raw results for the C excitonic feature as well as the α and β van Hove features, from which Figure 5, Figure 6 and Figure 7 have been derived.
